# Efficacy and Safety of Once-Weekly Insulin Regimes on Glycemic Control for Type 2 Diabetes: A Systematic Review and Network Meta-analysis

**DOI:** 10.1186/s13098-023-01240-5

**Published:** 2024-01-03

**Authors:** Peng Wang, Yu Zhang, Wenhao Xu, Jialing He, Liyuan Peng, Yuning Feng, Ping Xu, Weelic Chong, Yang Hai, Lu Jia, Fang Fang

**Affiliations:** 1 West China Hospital, Sichuan University, No. 37, Guo Xue Xiang, Chengdu, 610041 Sichuan China; 2 Center for Evidence-based Medicine, Affiliated Hospital of Chengdu University, Chengdu, Sichuan China; 3 Department of Surgical Intensive care medicine, Shanxi Provincial People’s Hospital, Taiyuan, Shanxi China; 4 Department of critical care medicine, Affiliated Hospital of Chengdu University, Chengdu, Sichuan China; 5 Department of Medical Oncology, Thomas Jefferson University, Philadelphia, PA USA; 6 Sidney Kimmel Medical College, Thomas Jefferson University, Philadelphia, PA USA; 7 Sichuan University Library, Chengdu, Sichuan China

**Keywords:** Insulin, Type 2 Diabetes, Icodec, Meta-analysis

## Abstract

**Background:**

Randomized controlled trials have found that once-weekly insulin resulted in greater glycemic control compared to once-daily insulin in patients with type 2 diabetes. However, no direct comparisons have been made between different types of once-weekly insulin thus far. This systematic review and network meta-analysis aimed to evaluate the effect of the two most advanced once-weekly insulin analogues, namely insulin icodec and insulin Fc, in patients with type 2 diabetes.

**Methods:**

We conducted a thorough search in the databases PubMed, Embase, and the Cochrane Central Register of Controlled Trials. The search included articles published from the beginning to October 10, 2023, with no language limitations. Our aim was to conduct a systematic review of randomized controlled trials that investigated the effectiveness and safety of once-weekly insulin in individuals with type 2 diabetes. Our primary outcome was to evaluate excellent glycemic control, defined as patients achieving glycated hemoglobin levels below 7%.

**Results:**

We identified a total of 7 trials involving 2829 patients. The results showed that once-weekly insulin icodec is more effective than once-weekly insulin Fc (RR 1.59 [95% CI 1.08–2.38]), once-daily degludec (RR 1.43 [95% CI 1.14–1.83]), and once-daily glargine (RR 1.15 [95% CI 1.00-1.41]). Moreover, the incidence of severe hypoglycemia was lower with once-weekly insulin icodec compared to once-daily degludec (RR 0.00016 [95% CI 0 to 0.41]). However, no significant difference in the incidence of severe hypoglycemia was observed between once-weekly insulin icodec and once-daily glargine (RR 0.39 [95% CI 0.03 to 4.83]).

**Conclusions:**

In patients with type 2 diabetes, once-weekly insulin icodec achieved superior glycemic control compared to once-weekly insulin Fc, with no significant difference in the occurrence of hypoglycemia. The ranking probability results have shown that one weekly icodec seems to be the preferred option in patients with type 2 diabetes.

**Trial registration:**

PROSPERO Identifier: CRD42023470894.

**Supplementary Information:**

The online version contains supplementary material available at 10.1186/s13098-023-01240-5.

## Introduction

Type 2 diabetes poses a significant health burden worldwide, requiring effective management strategies to control glycemic levels. When noninsulin glucose-lowering agents fail to achieve optimal glycemic control, many individuals with type 2 diabetes resort to basal insulin treatment [1]. However, the daily administration of basal insulins may present challenges in terms of treatment adherence, persistence, and treatment burden.

In order to address these challenges, individuals with type 2 diabetes have expressed a strong preference for a once-weekly injectable treatment option. Such an option has the potential to improve treatment adherence, enhance persistence, and reduce the overall treatment burden. To fulfill this need, two advanced basal insulin analogues have been developed specifically for once-weekly subcutaneous administration in individuals with diabetes. The first option is insulin icodec, which is an insulin analog acylated with a C20 fatty diacid (icosanedioic acid) side chain. The second option is insulin Fc, which is a fusion protein combining a single-chain insulin variant with a human immunoglobulin G fragment crystallizable domain [2].

To evaluate the effectiveness of these once-weekly insulin options, several randomized controlled trials have been conducted. For instance, the ONWARDS trial found that once-weekly insulin resulted in greater glycemic control compared to once-daily insulin [3–7]. However, it is noteworthy that no direct comparisons have been made between different types of once-weekly insulin thus far.

Considering the lack of direct evidence, network meta-analysis is an increasingly employed statistical methodology that facilitates the estimation of comparative treatment effectiveness [8, 9]. Therefore, the objective of this study is to conduct a systematic review and network meta-analysis to evaluate the effect of the two most advanced once-weekly insulin analogues, namely insulin icodec and insulin Fc, in patients with type 2 diabetes.

## Methods

### Protocol and guidance

The study followed the standard guidelines provided by the reporting of systematic reviews and network meta-analysis [10, 11]. Additionally, the study protocol was registered with PROSPER (CRD42023470894).

### Criteria for considering studies for this review

The eligibility criteria for this study were determined based on the PICOS Criteria (participants, interventions, comparators, outcomes, and study design). We included published randomized controlled trials that met the following criteria:

1. Population: The study included adults (age ≥ 18) diagnosed with type 2 diabetes, whether they have used insulin in the past.
2. Intervention: We included studies that investigated once-weekly insulin, including but not limited to insulin icodec and insulin Fc (IF, insulin efsitora alfa). Insulin could have been administered regardless of the type, dose, or duration. If other treatment medications were given, they had to be the same in all groups.
3. Comparison intervention: once-daily insulin, including but not limited to insulin glargine and insulin degludec.
4. Outcome: The primary outcome was the proportion of patients achieving HbA1c levels below 7%. Secondary safety outcomes included hypoglycemia alerts episodes (hypoglycemia confirmed by glucose level < 70 mg/dL or ≥ 54 mg /dL, clinically significant hypoglycemia (hypoglycemia confirmed by glucose level < 54 mg/dL, severe hypoglycemia (hypoglycemia associated with severe cognitive impairment requiring external assistance for recovery), any adverse event, serious adverse event and any injection-site reaction.
5. Study design: We included randomized controlled trials but excluded crossover trials.

### Search strategy

Our research team conducted a comprehensive literature search using the PubMed, Embase, and Cochrane Central Register of Controlled Trials (CENTRAL) databases until October 10, 2023, without any language restrictions. We also searched ClinicalTrials.gov and the World Health Organization International Clinical Trials Registry Platform to identify ongoing or unpublished trials that fulfilled the potential eligibility criteria. To ensure a comprehensive search for relevant articles, we manually reviewed the reference lists of identified trials and systematic reviews. For more information on our search strategy, please refer to Table S1 in the supplement.

### Study selection

Two reviewers (PW and WX) independently evaluated the relevance of titles and abstracts after removing duplicate studies. The full texts of selected articles were obtained to determine their eligibility for inclusion. Any disagreements between the reviewers were resolved through consensus or by consulting a third reviewer (YZ).

### Data collection process

Two reviewers (PW and WX) employed a standardized form for data extraction from the included trials independently. Data were extracted based on intention-to-treat principles, whereby all randomized participants contributed data based on their assigned treatment. When relevant information for the outcome could not be extracted from published reports, we additionally reached out to the corresponding authors via email to acquire any necessary missing data. We resolved discrepancies either through consensus or by inviting a third reviewer (YZ) for assistance.

### Quality assessment

Two independent reviewers (PW and WX) assessed the risk of bias in the included trials using the Risk of Bias tool, [12] which consists of five domains. Each trial was assigned a study-level score for each domain, indicating the level of bias risk as low, high, or some concerns. The certainty of evidence in the meta-analysis was assessed using the Grading of Recommendations, Assessment, Development, and Evaluation (GRADE) methodology, based on established guidelines [13]. Any disagreements in the assessments were resolved through consensus or by inviting a third reviewer (YZ) to make the final decision in cases where consensus could not be reached.

### Data synthesis

This network meta-analysis was conducted using R software (version 4.3.1), specifically the netmeta and gemtc packages (version 1.0–1), which employ both Bayesian and frequentist approaches. The goal of this analysis was to compare multiple treatments using the Markov chain Monte Carlo method with vague priors [14].

To achieve model convergence, we utilized generalized linear models with 4 chains and 50,000 iterations for each chain. The first 50,000 iterations were considered as burn-in and discarded, while the subsequent 100,000 iterations were used to ensure model convergence. We assessed model convergence by evaluating Gelman-Rubin plots, utilizing the potential scale reduction factor as an indicator. A potential scale reduction factor close to one indicated complete convergence of the model. If both direct and indirect evidence were available for a specific pairwise comparison, we assessed their agreement using methods to identify inconsistencies in the network meta-analysis.

For dichotomous variables, treatment effects were estimated using the risk ratio (RR) with a 95% confidence interval [15]. We utilized the Netleague command to report the relative treatment effects for all pairwise comparisons estimated through network meta-analysis. Additionally, we ranked the interventions based on the primary outcome using a Rankogram plot, which displays the probability of interventions being ranked in various positions [16].

## Results

### Included studies and study characteristics

**Fig. 1:**
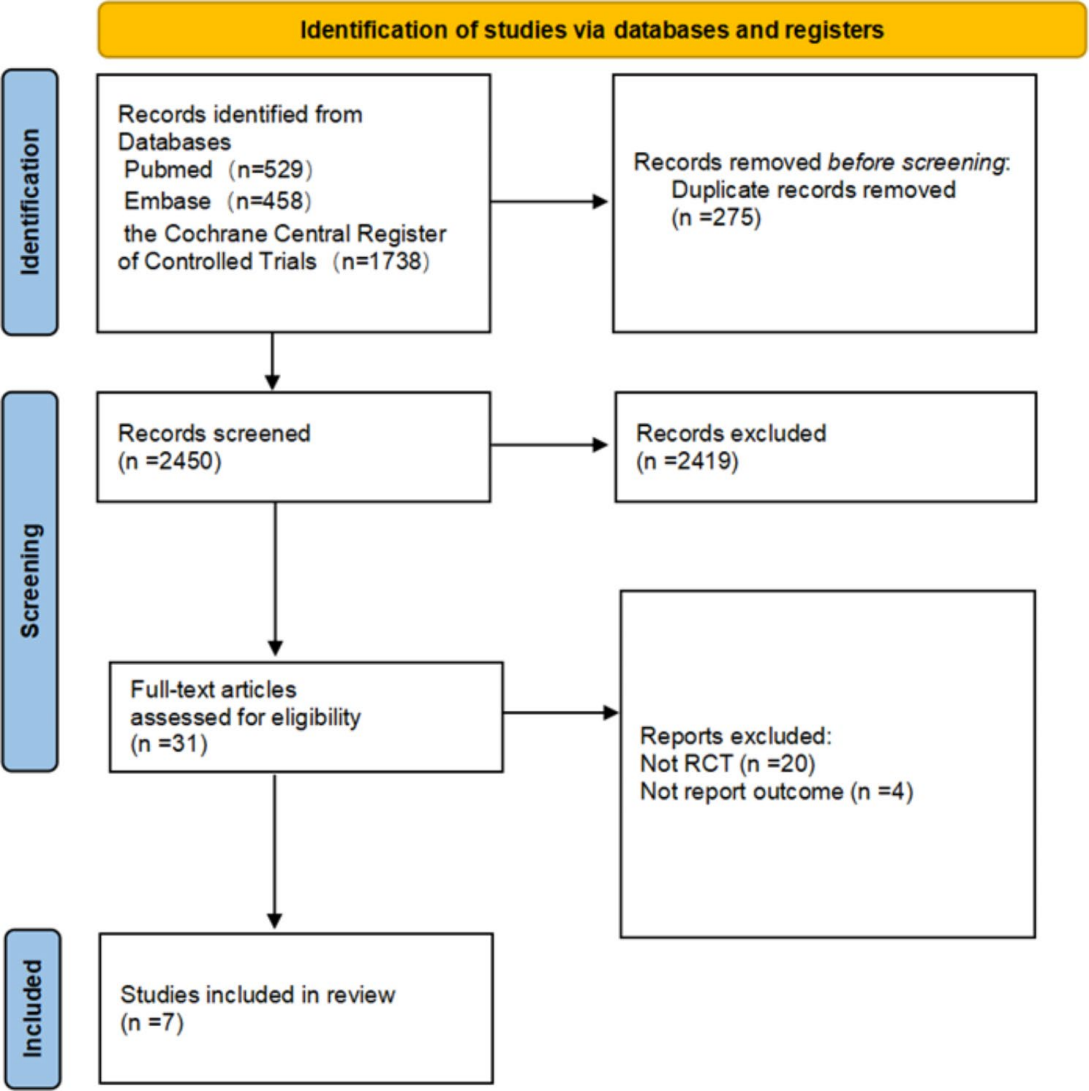
Search strategy and final included and excluded studies

Figure 1 presents the PRISMA diagram depicting the process of the meta-analysis. Our comprehensive electronic literature search initially identified a total of 2725 studies. Ultimately, 7 trials [4, 6, 7, 17–20] met the eligibility criteria and were included in the meta-analysis.

The main characteristics of the included trials are shown in Table 1. The studies included in the analysis were published from 2020 to 2023 and encompassed a total of 2829 patients. The sample sizes varied from 102 to 984. The median duration of follow-up was 26 weeks, ranging from 16 to 78 weeks.

**Table 1:** Characteristics of included studies

Study	Patients, n	Male, (%)	Age, years	Intervention	Control	Follow-up
Rosenstock et al. 2020	247	56.3	59.6	Once-weekly insulin icodec	Once-daily glargine	26 weeks
Bajaj et al. 2021	104	72.1	61.7	Once-weekly insulin icodec	Once-daily glargine	16 weeks
Lingvay et al. 2021	102	53.9	60.7	Once-weekly insulin icodec + non-insulin glucose-lowering agents	Once-daily glargine + non-insulin glucose-lowering agents	16 weeks
Lingvay et al. 2023	588	62.8	59.0	Once-weekly insulin icodec + non-insulin glucose-lowering agents + once-daily placebo	Once-daily degludec + non-insulin glucose-lowering agents + once-weekly placebo	26 weeks
Philis-Tsimikas et al. 2023	526	57.4	62.5	Once-weekly insulin icodec ± non-insulin glucose-lowering agents	Once-daily degludec ± non-insulin glucose-lowering agents	26 weeks
Rosenstock et al. 2023	984	56.7	59.0	Once-weekly insulin icodec + non-insulin glucose-lowering agents	Once-daily glargine + non-insulin glucose-lowering agents	78 weeks
Bue-Valleskey et al. 2023	278	54.7	58.4	Once-weekly insulin Fc	Once-daily insulin degludec	26 weeks

### Risk-of-bias assessments

Risk-of-bias assessments are presented in Supplement Fig. 1. The analysis identifies that 3 trials [4, 17, 20] were identified as having a low risk of bias, 1 trial [6] was identified as a unclear risk of bias and the remaining 3 trials [7, 18, 19] were deemed to have a high risk of bias, mainly because some trials employed an open-label design; However, the continuous glucose monitoring recordings used for analyzing the primary endpoint were blinded from both the investigators and the trial participants.

### The primary outcome

**Fig. 2:**
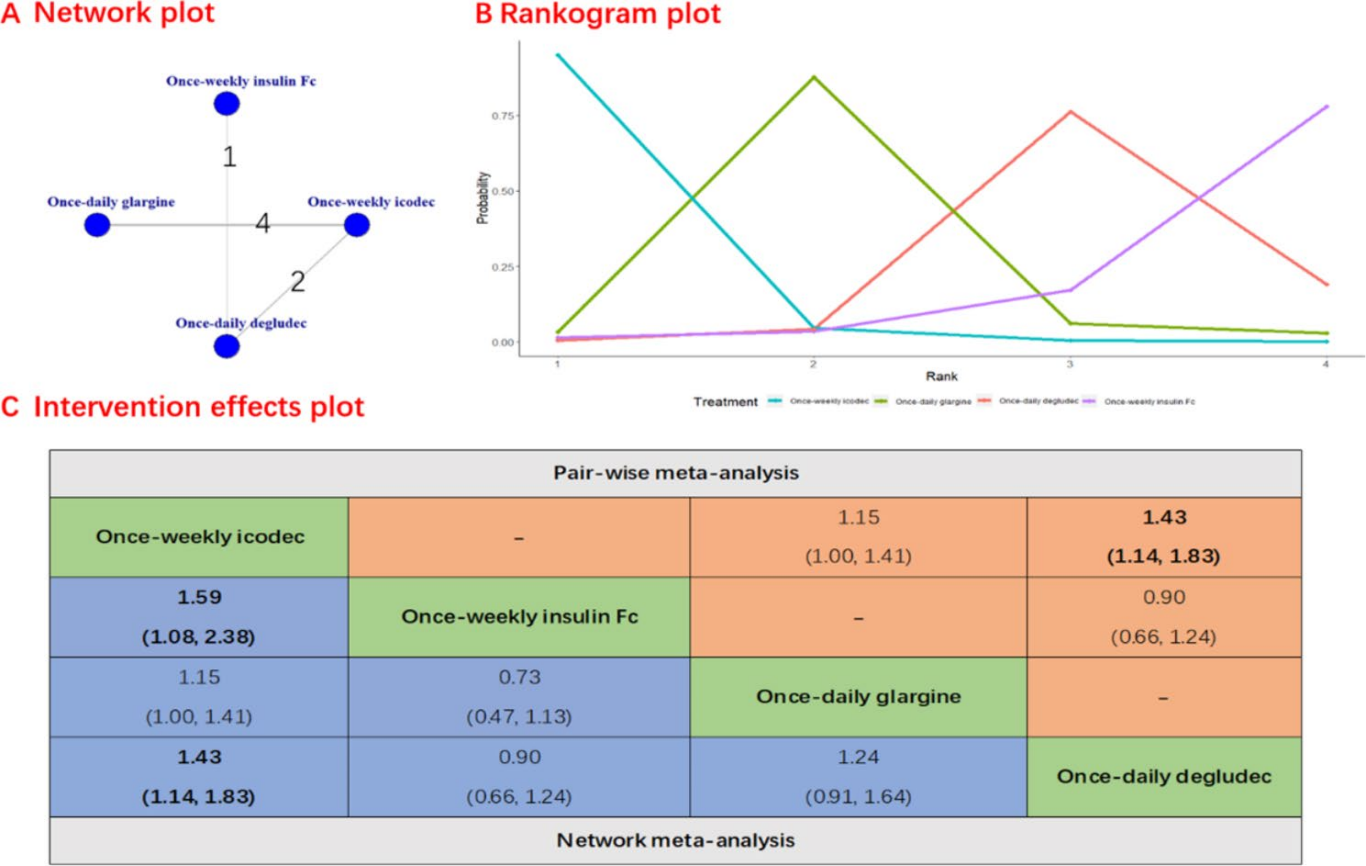
Forest plots of once-weekly insulin icodec versus once-weekly insulin FC for HbA1c levels below 7%

A total of 7 studies, involving 2829 patients, reported the proportion of patients achieving HbA1c levels below 7%. Figure 2 presents the results of the Network plot, rankogram plot and intervention effects plot (both pairwise meta-analyses and network meta-analysis) for the proportion of patients achieving HbA1c levels below 7%. There was evidence to support the notion that once-weekly insulin icodec is more effective than once-weekly insulin Fc (RR, 1.59; 95% CI, 1.08–2.38), once-daily degludec (RR, 1.43; 95% CI, 1.14–1.83) and once-daily glargine (RR, 1.15; 95% CI, 1.00-1.41). Notably, the rankogram plot indicated that once-weekly insulin icodec had the highest statistical probability of being the optimal choice for the proportion of patients achieving HbA1c levels below 7% (Fig. 2). once-weekly insulin icodec had the highest SUCRA value 0.95, followed by once-daily glargine (SUCRA 0.88), once -daily degludec (SUCRA, 0.76) and once-insulin Fc (SUCRA, 0.78). The potential scale reduction factor value of 1.000 suggested a strong iterative effect, complete convergence, and stable model outputs. The S2 table presented the main findings of the GRADE assessment of certainty for the outcome.

### Secondary efficacy outcomes

**Fig. 3:**
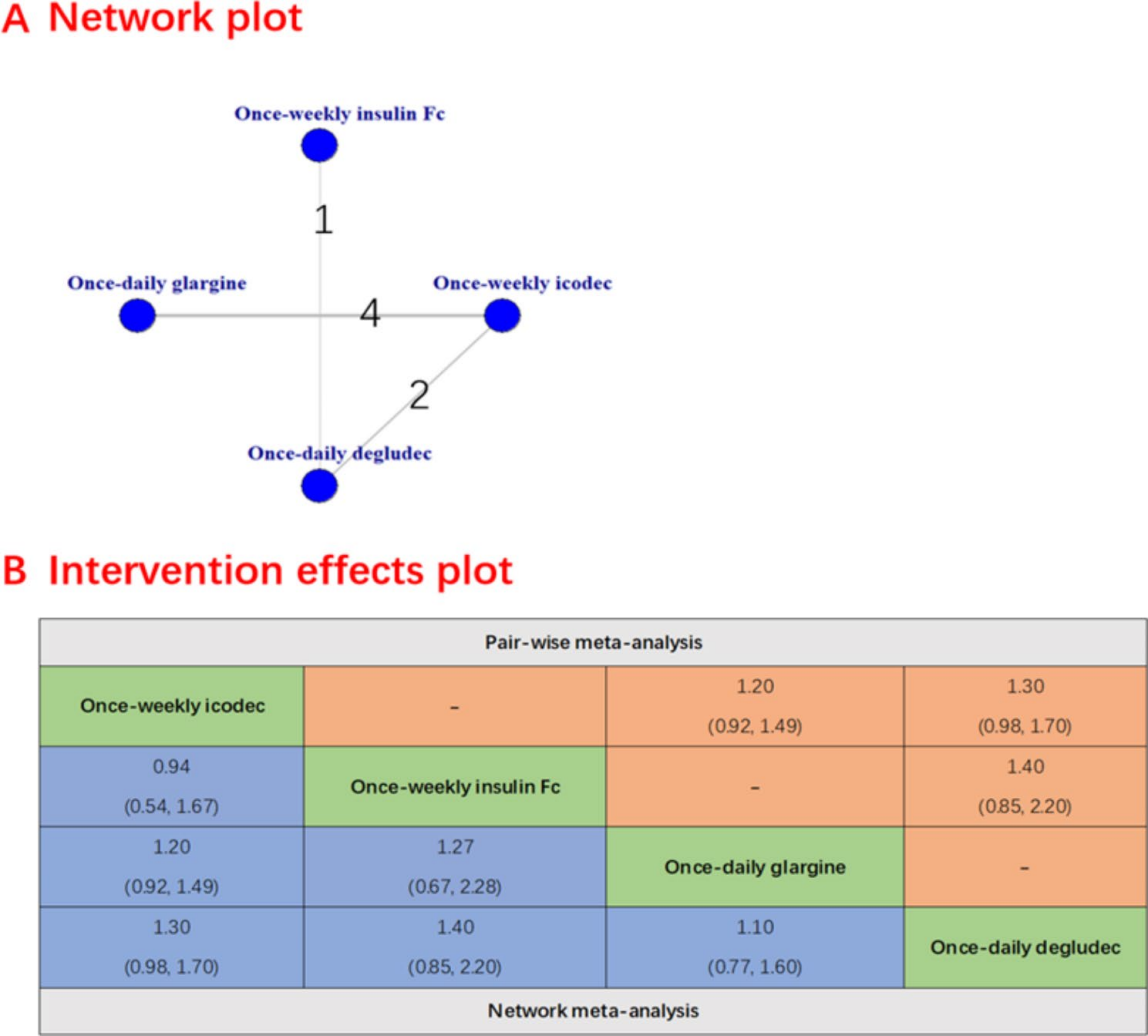
Forest plots of once-weekly insulin icodec versus once-weekly insulin FC for severe hypoglycemia

**Fig. 4:**
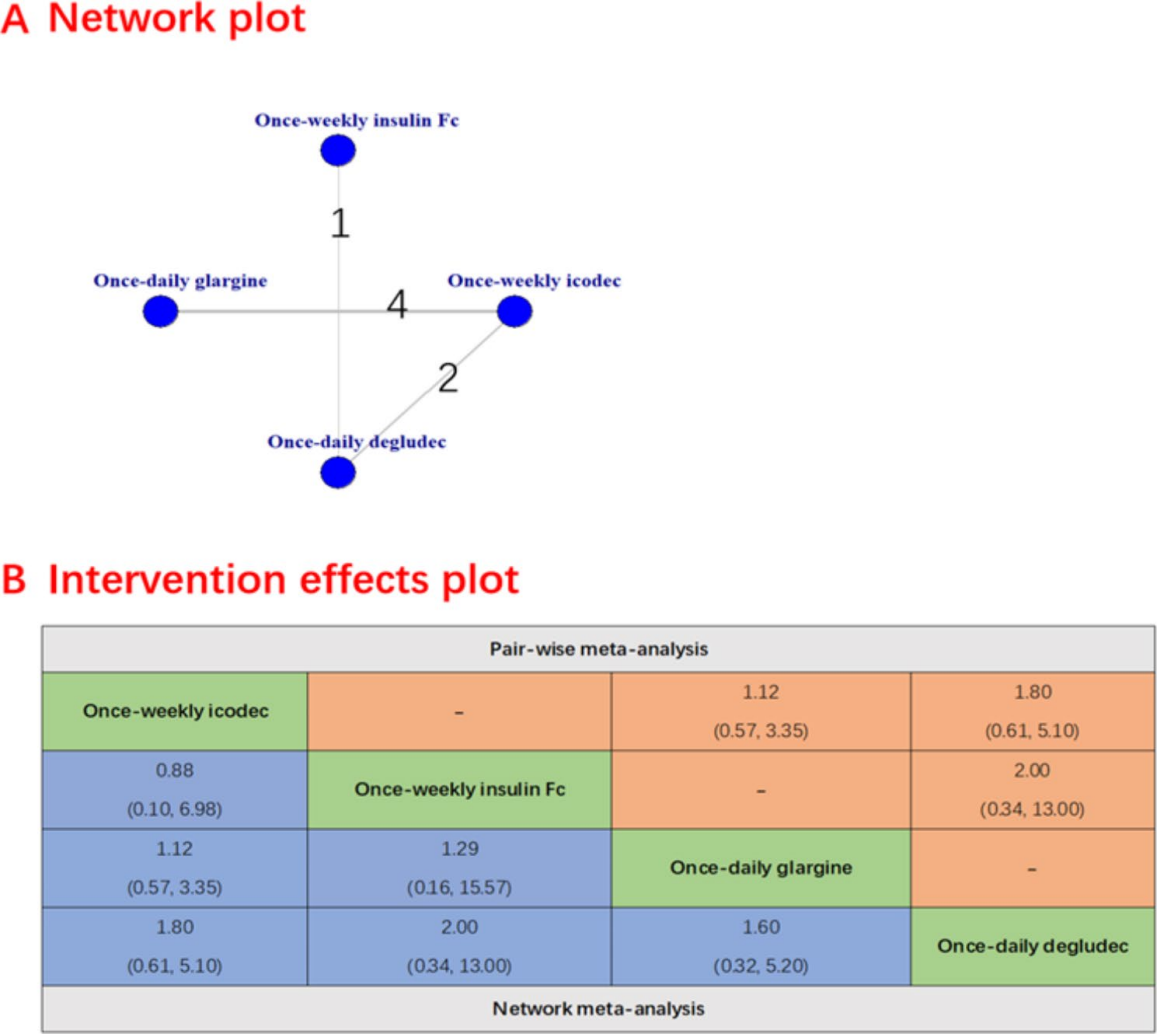
Forest plots of once-weekly insulin icodec versus once-weekly insulin FC for hypoglycemia alerts episodes

**Fig. 5:**
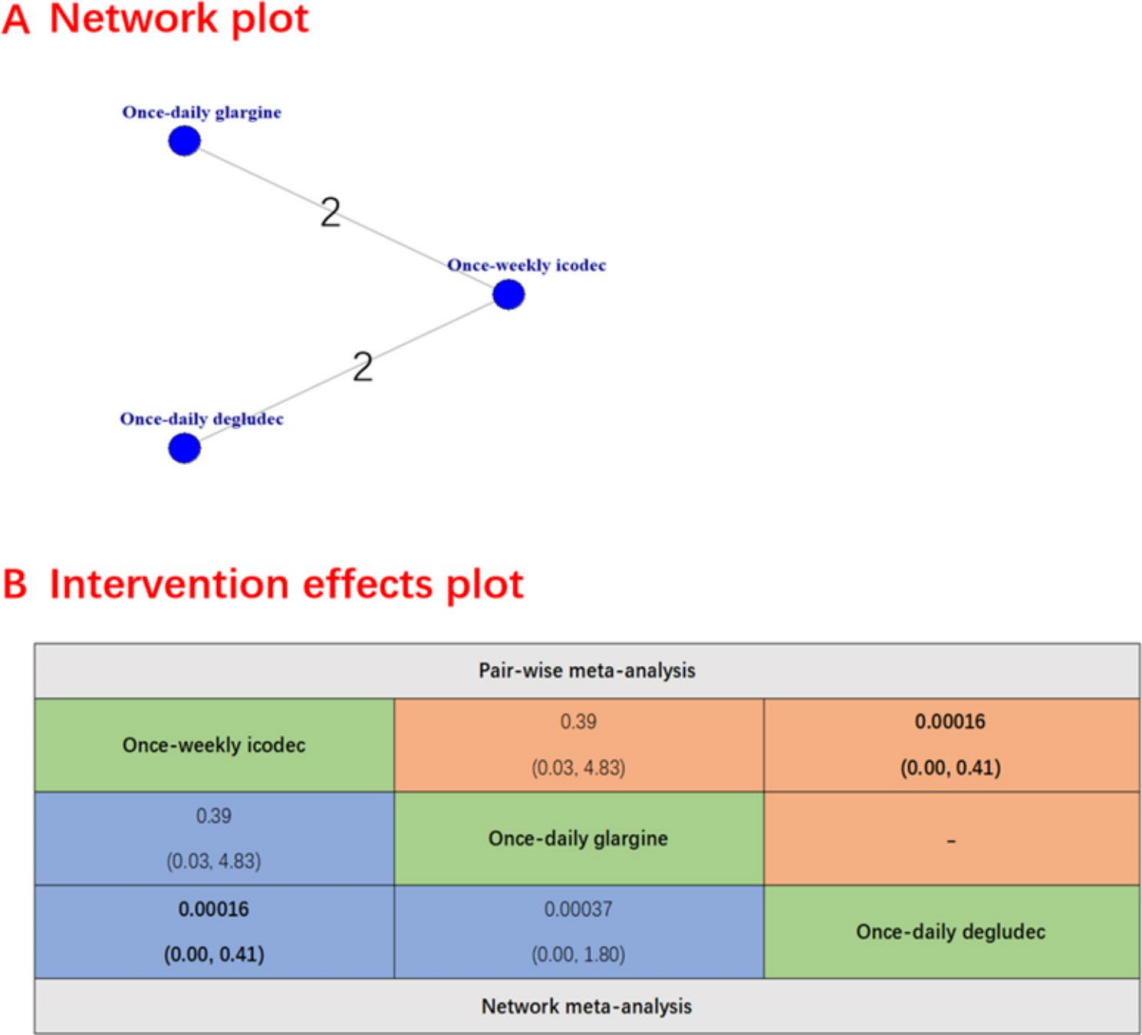
Forest plots of once-weekly insulin icodec versus once-weekly insulin FC for clinically significant hypoglycemia

The secondary safety outcomes were evaluated and presented. The once-weekly insulin icodec was associated with a lower incidence of severe hypoglycemia compared to the once-daily deglude (RR 0.00016 [95% CI 0 to 0.41], Fig. 3) and no significant difference was observed between once-weekly insulin icodec and once-daily glargine (RR 0.39 [95% CI 0.03 to 4.83]).There were no significant differences between once-weekly insulin icodec and once-weekly insulin Fc (RR 0.94 [95% CI 0.54 to 1.67]), once-daily glargine (RR 1.20 [95% CI 0.92 to 1.49) and once-daily deglude (RR 1.30 [95% CI 0.98 to 1.70]) in terms of hypoglycemia alerts episodes (Fig. 4). Similarly, there was also no significant difference between once-weekly insulin icodec and once-weekly insulin Fc, once-daily glargin and once-daily deglude including clinically significant hypoglycemia (Fig. 5), any adverse event (S2 Figure), serious adverse event (S3 Figure) and any injection-site reaction (S4 Figure).

## Discussion

This study found that treatment with once-weekly insulin icodec led to improved glycemic control compared to other insulin regimens, including once-weekly insulin FC and once-daily insulin. Importantly, there was no significant difference in the incidence of hypoglycemia between the once-weekly and once-daily insulin groups, indicating similar safety profiles. In summary, these findings suggest that once-weekly insulin icodec may be a viable and safe treatment option for patients with type 2 diabetes.

### Comparison with other studies

These results are consistent with previous randomized controlled trials and meta-analyses [21, 22] that have shown the superiority of once-weekly insulin over once-daily insulin in improving glycemic control in patients with type 2 diabetes. However, no trials have compared outcomes after the use of once-weekly insulin icodec and other once-weekly insulin regimes. Karakasis et al. [21] conducted a meta-analysis to assess the effect of once-weekly insulin in type 2 diabetes patients. The results of subgroup analysis, based on different types of once-weekly insulin, suggest that once-weekly insulin icodec may be a more preferable choice compared to other alternatives. Our study adds value by comparing different types of once-weekly insulin analogues, providing insights into their relative effectiveness. The significantly higher efficacy of once-weekly insulin icodec compared to once-weekly insulin Fc suggests that it may be a preferred option for achieving excellent glycemic control in patients with type 2 diabetes.

Regarding the safety profile, we found that the risk of adverse events associated with once-weekly insulin icodec is similar to that of other insulin regimes. This means that the improved effectiveness of once-weekly insulin icodec compared to other insulin regimes is not accompanied by a higher risk of adverse events.

The variations in pharmacokinetics and pharmacodynamics of these insulin types likely contribute to these findings. Insulin icodec, as a long-acting insulin, provides sustained release over a week, leading to more stable blood glucose levels [23]. In contrast, insulin Fc, a short-acting insulin, requires daily administration and may result in greater fluctuations in blood glucose control. Our findings underscore the importance of considering the specific characteristics of different insulin formulations when evaluating their effectiveness in glycemic control. Further research, including randomized controlled trials directly comparing these two formulations, is necessary to confirm these conclusions and provide stronger evidence.

### Strengths and limitations

This review demonstrates several strengths, including a comprehensive search for evidence, the use of an a priori protocol, and the assessment of eligibility, risk of bias, and data abstraction by multiple reviewers. Moreover, the evaluation of the quality of evidence in this review was conducted meticulously, resulting in the identification of high-quality evidence for numerous critical outcomes. The meta-analysis included an extensive search of relevant databases without language restrictions, thereby increasing the likelihood of capturing relevant studies. The primary outcome measure, excellent glycemic control, is commonly utilized and holds clinical relevance in the management of patients with type 2 diabetes, defined as achieving a HbA1c level below 7%.

This network meta-analysis still has some limitations. Firstly, the duration of follow-up and the variation in titration algorithms among the identified randomized controlled trials introduce heterogeneity and may limit the generalizability of our findings. Secondly, in addition, the open-label design adopted in most randomized controlled trials, motivated by safety considerations, may have influenced dose adjustments as well as the reporting and monitoring of adverse events. Furthermore, it is worth noting that the trials included in this meta-analysis primarily examined intermediate and short-term outcomes. Consequently, the long-term impact of once-weekly insulin on the prevention of cardiovascular events and mortality remains uncertain. To address this knowledge gap and establish the durability of the observed beneficial effects, further studies with extended follow-up durations are warranted.

### Implications

It’s important to note that guidelines regarding once-weekly insulin are still in development. The American Diabetes Association (ADA) and the European Association for the Study of Diabetes (EASD) [24] have not yet included recommendations specific to this insulin formulation in their guidelines. However, as more research is conducted and more clinical experience is gained, these guidelines may be updated to reflect the potential benefits and appropriate use of once-weekly insulin icodec. Our findings have important implications for both professionals and policymakers in the field of diabetes treatment. The use of once-weekly insulin especially the once-weekly insulin icodec may facilitate treatment acceptance and adherence among patients, as it requires less frequent administration and produces better glycemic control and similar safety. This can potentially improve patient outcomes and increase treatment satisfaction. Ultimately, the choice of once-weekly insulin icodec should be made on an individual basis, taking into consideration factors such as patient preferences, lifestyle, and goals of therapy. Close monitoring and consultation with a healthcare professional are vital to ensure optimal treatment outcomes for patients with diabetes.

Moving forward, further research is warranted to explore the long-term efficacy, safety profile, and cost-effectiveness of once-weekly insulin icodec compared to once-weekly insulin FC. Additionally, studying the impact of once-weekly insulin icodec on patient’s quality of life and satisfaction with treatment would provide valuable insights.

This study demonstrated that once-weekly insulin icodec achieved superior glycemic control in patients with type 2 diabetes compared to once-weekly insulin FC, without significant differences of hypoglycemia. The ranking probability results suggested that once-weekly insulin icodec may be the preferred treatment option for patients with type 2 diabetes.

### Electronic supplementary material

Below is the link to the electronic supplementary material.

Supplementary Material 1

Supplementary Material 2

Supplementary Material 3

## Data Availability

No additional data is available.

## Declarations

### Patient confidentiality forms

Not required.
