# Supplementary material for: Efficacy and Safety of Once-Weekly Insulin Regimes on Glycemic Control for Type 2 Diabetes: A Systematic Review and Network Meta-analysis
Source: Diabetol Metab Syndr. 2024 Jan 3;16:3. doi: 10.1186/s13098-023-01240-5 (PMC10763463; doi:10.1186/s13098-023-01240-5)
Supplement: Supplementary file 1 — Supplementary Material 1 [file 13098_2023_1240_MOESM1_ESM.docx]

**Supporting information**

**S1 Table. Search strategy**

**S2 Table. Results and certainty assessments for the primary outcome comparing once-weekly insulin with once-weekly Fc.**

**S1 Fig. Risk of bias summary**

**S2 Fig. Network plot and league plot of the network estimates for comparing once-weekly insulin with once-weekly Fc for the risk of any adverse event.**

**S3 Fig. Network plot and league plot of the network estimates for comparing once-weekly insulin with once-weekly Fc for the risk of serious adverse event.**

**S4 Fig. Network plot and league plot of the network estimates for comparing once-weekly insulin with once-weekly Fc for the risk of any injection-site reaction.**

**Supporting information**

**S1 Table. Search strategy**

| **PubMed** | | |
| --- | --- | --- |
|  | "Diabetes Mellitus, Type 2"[Mesh] | 173275 |
|  | ((((((((((((((((((((((((((((((Diabetes Mellitus, Noninsulin-Dependent[Title/Abstract]) OR (Diabetes Mellitus, Ketosis-Resistant[Title/Abstract])) OR (Diabetes Mellitus, Ketosis Resistant[Title/Abstract])) OR (Ketosis-Resistant Diabetes Mellitus[Title/Abstract])) OR (Diabetes Mellitus, Non Insulin Dependent[Title/Abstract])) OR (Diabetes Mellitus, Non-Insulin-Dependent[Title/Abstract])) OR (Non-Insulin-Dependent Diabetes Mellitus[Title/Abstract])) OR (Diabetes Mellitus, Stable[Title/Abstract])) OR (Stable Diabetes Mellitus[Title/Abstract])) OR (Diabetes Mellitus, Type II[Title/Abstract])) OR (NIDDM[Title/Abstract])) OR (Diabetes Mellitus, Noninsulin Dependent[Title/Abstract])) OR (Diabetes Mellitus, Maturity-Onset[Title/Abstract])) OR (Diabetes Mellitus, Maturity Onset[Title/Abstract])) OR (Maturity-Onset Diabetes Mellitus[Title/Abstract])) OR (Maturity Onset Diabetes Mellitus[Title/Abstract])) OR (MODY[Title/Abstract])) OR (Diabetes Mellitus, Slow-Onset[Title/Abstract])) OR (Diabetes Mellitus, Slow Onset[Title/Abstract])) OR (Slow-Onset Diabetes Mellitus[Title/Abstract])) OR (Type 2 Diabetes Mellitus[Title/Abstract])) OR (Noninsulin-Dependent Diabetes Mellitus[Title/Abstract])) OR (Noninsulin Dependent Diabetes Mellitus[Title/Abstract])) OR (Maturity-Onset Diabetes[Title/Abstract])) OR (Diabetes, Maturity-Onset[Title/Abstract])) OR (Maturity Onset Diabetes[Title/Abstract])) OR (Type 2 Diabetes[Title/Abstract])) OR (Diabetes, Type 2[Title/Abstract])) OR (Diabetes Mellitus, Adult-Onset[Title/Abstract])) OR (Adult-Onset Diabetes Mellitus[Title/Abstract])) OR (Diabetes Mellitus, Adult Onset[Title/Abstract]) | 183700 |
|  | (#1) OR (#2) | 236206 |
|  | "Insulin"[Mesh] | 199868 |
|  | ((((((((((Insulin, Regular[Title/Abstract]) OR (Regular Insulin[Title/Abstract])) OR (Soluble Insulin[Title/Abstract])) OR (Insulin, Soluble[Title/Abstract])) OR (Insulin A Chain[Title/Abstract])) OR (Sodium Insulin[Title/Abstract])) OR (Insulin, Sodium[Title/Abstract])) OR (Novolin[Title/Abstract])) OR (Iletin[Title/Abstract])) OR (Insulin B Chain[Title/Abstract])) OR (Chain, Insulin B[Title/Abstract]) | 2569 |
|  | (#4) OR (#5) | 200544 |
|  | (Once-Weekly[Title/Abstract]) OR (week[Title/Abstract]) | 593653 |
|  | ((Once-Daily[Title/Abstract]) OR (Daily[Title/Abstract])) OR (Day[Title/Abstract]) | 1749657 |
|  | "Randomized Controlled Trials as Topic"[Mesh] | 168174 |
|  | ((((randomized controlled trial[Title/Abstract]) OR (controlled clinical trial[Title/Abstract])) OR (randomized[Title/Abstract])) OR (randomly[Title/Abstract])) OR (trial[Title/Abstract]) | 1440422 |
|  | (#9) OR (#10) | 1504145 |
|  | ((((#3) AND (#6)) AND (#7)) AND (#8)) AND (#11) | 529 |
| **EMBASE** | | |
|  | ('diabetes'exp OR diabetes) AND mellitus, AND type AND ('2'exp OR 2) | 422891 |
|  | 'diabetes mellitus, noninsulin-dependent'ab,ti OR 'diabetes mellitus, ketosis-resistant'ab,ti OR 'diabetes mellitus, ketosis resistant'ab,ti OR 'ketosis-resistant diabetes mellitus'ab,ti OR 'diabetes mellitus, non insulin dependent'ab,ti OR 'diabetes mellitus, non-insulin-dependent'ab,ti OR 'non-insulin-dependent diabetes mellitus'ab,ti OR 'diabetes mellitus, stable'ab,ti OR 'stable diabetes mellitus'ab,ti OR 'diabetes mellitus, type ii'ab,ti OR 'niddm'ab,ti OR 'diabetes mellitus, noninsulin dependent'ab,ti OR 'diabetes mellitus, maturity-onset'ab,ti OR 'diabetes mellitus, maturity onset'ab,ti OR 'maturity-onset diabetes mellitus'ab,ti OR 'maturity onset diabetes mellitus'ab,ti OR 'mody'ab,ti OR 'diabetes mellitus, slow-onset'ab,ti OR 'diabetes mellitus, slow onset'ab,ti OR 'slow-onset diabetes mellitus'ab,ti OR 'type 2 diabetes mellitus'ab,ti OR 'noninsulin-dependent diabetes mellitus'ab,ti OR 'noninsulin dependent diabetes mellitus'ab,ti OR 'maturity-onset diabetes'ab,ti OR 'diabetes, maturity-onset'ab,ti OR 'maturity onset diabetes'ab,ti OR 'type 2 diabetes'ab,ti OR 'diabetes, type 2'ab,ti OR 'diabetes mellitus, adult-onset'ab,ti OR 'adult-onset diabetes mellitus'ab,ti OR 'diabetes mellitus, adult onset'ab,ti | 270978 |
|  | #1 OR #2 | 453143 |
|  | 'insulin' | 984639 |
|  | 'insulin, regular'ab,ti OR 'regular insulin'ab,ti OR 'soluble insulin'ab,ti OR 'insulin, soluble'ab,ti OR 'insulin a chain'ab,ti OR 'sodium insulin'ab,ti OR 'insulin, sodium'ab,ti OR 'novolin'ab,ti OR 'iletin'ab,ti OR 'insulin b chain'ab,ti OR 'chain, insulin b'ab,ti | 3477 |
|  | #4 OR #5 | 984639 |
|  | 'once-weekly'ab,ti OR 'week'ab,ti | 918372 |
|  | 'once-daily'ab,ti OR 'daily'ab,ti | 940584 |
|  | 'randomized controlled trial (topic)' | 263117 |
|  | 'randomized controlled trial'ab,ti OR 'controlled clinical trial'ab,ti OR 'randomi'ti | 161435 |
|  | #9 OR #10 | 411936 |
|  | #3 AND #6 AND #7 AND #8 AND #11 | 458 |
| COCHRANE CENTRAL | | |
|  | Diabetes Mellitus, Type 2 | 68840 |
|  | (Diabetes Mellitus, Noninsulin-Dependent):ab,ti,kw OR(Diabetes Mellitus, Ketosis-Resistant):ab,ti,kw OR(Diabetes Mellitus, Ketosis Resistant):ab,ti,kw OR(Ketosis-Resistant Diabetes Mellitus):ab,ti,kw OR(Diabetes Mellitus, Non Insulin Dependent):ab,ti,kw OR(Diabetes Mellitus, Non-Insulin-Dependent):ab,ti,kw OR(Non-Insulin-Dependent Diabetes Mellitus):ab,ti,kw OR(Diabetes Mellitus, Stable):ab,ti,kw OR(Stable Diabetes Mellitus):ab,ti,kw OR(Diabetes Mellitus, Type II):ab,ti,kw OR(NIDDM):ab,ti,kw OR(Diabetes Mellitus, Noninsulin Dependent):ab,ti,kw OR(Diabetes Mellitus, Maturity-Onset):ab,ti,kw OR(Diabetes Mellitus, Maturity Onset):ab,ti,kw OR(Maturity-Onset Diabetes Mellitus):ab,ti,kw OR(Maturity Onset Diabetes Mellitus):ab,ti,kw OR(MODY):ab,ti,kw OR(Diabetes Mellitus, Slow-Onset):ab,ti,kw OR(Diabetes Mellitus, Slow Onset):ab,ti,kw OR(Slow-Onset Diabetes Mellitus):ab,ti,kw OR(Type 2 Diabetes Mellitus):ab,ti,kw OR(Noninsulin-Dependent Diabetes Mellitus):ab,ti,kw OR(Noninsulin Dependent Diabetes Mellitus):ab,ti,kw OR(Maturity-Onset Diabetes):ab,ti,kw OR(Diabetes, Maturity-Onset):ab,ti,kw OR(Maturity Onset Diabetes):ab,ti,kw OR(Type 2 Diabetes):ab,ti,kw OR(Diabetes, Type 2):ab,ti,kw OR(Diabetes Mellitus, Adult-Onset):ab,ti,kw OR(Adult-Onset Diabetes Mellitus):ab,ti,kw OR(Diabetes Mellitus, Adult Onset):ab,ti,kw | 62556 |
|  | #1 OR #2 | 78048 |
|  | Insulin | 73660 |
|  | (Insulin, Regular):ab,ti,kw OR(Regular Insulin):ab,ti,kw OR(Soluble Insulin):ab,ti,kw OR(Insulin, Soluble):ab,ti,kw OR(Insulin A Chain):ab,ti,kw OR(Sodium Insulin):ab,ti,kw OR(Insulin, Sodium):ab,ti,kw OR(Novolin):ab,ti,kw OR(Iletin):ab,ti,kw OR(Insulin B Chain):ab,ti,kw OR(Chain, Insulin B):ab,ti,kw | 8148 |
|  | #4 OR #5 | 73662 |
|  | (Once-Weekly):ab,ti,kw OR(week):ab,ti,kw | 274875 |
|  | (Once-Daily):ab,ti,kw OR(Daily):ab,ti,kw in Trials | 219631 |
|  | Randomized Controlled Trials as Topic | 82072 |
|  | (randomized controlled trial):ab,ti,kw OR(controlled clinical trial):ab,ti,kw OR(randomi*):ab,ti,kw | 1180284 |
|  | #9 OR #10 | 1181224 |
|  | #3 AND #6 AND #7 AND #8 AND #11 with Publication Year from 2015 to 2023, with Cochrane Library publication date Between Jan 2015 and Jan 2023, in Trials | 1738 |

**S2 Table. Results and certainty assessments for the primary outcome comparing once-weekly insulin with once-weekly Fc.**

| **Comparison** | Direct estimate | Network  estimate | Absolute Difference^*^  (95% CI) | GRADE |
| --- | --- | --- | --- | --- |
| Once-weekly icodec vs. Once-weekly insulin Fc | - | 1.59(1.08,2.38) | 8(1, 19) | Moderate^#^ |
| Once-weekly icodec vs. Once-daily glargine | 1.15(1.00,1.41) | 1.15(1.00,1.41) | 9(0, 24) | High |
| Once-weekly icodec vs. Once-daily degludec | 1.43(1.14,1.83) | 1.43(1.14,1.83) | 11(4, 22) | High |
| Once-weekly insulin Fc vs. Once-daily glargine | - | 0.73(0.47,1.13) | -15(-30,7) | Moderate^#^ |
| Once-weekly insulin Fc vs. Once-daily degludec | 0.90(0.66,1.24) | 0.90(0.66,1.24) | -3(-9,6) | Moderate^#^ |
| Once-daily glargine vs. Once-daily degludec | - | 1.24(0.91,1.64) | 6 (-2,17) | High |

*****Absolute Effect Estimates, No. Of Patients Per 1000

# Imprecisions

**S1 Fig. Risk of bias summary**

**
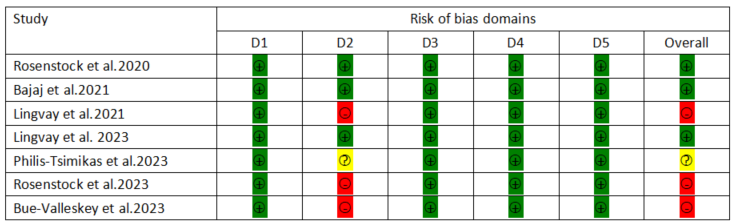
**

Domains:

D1: Bias arising from the randomization process.

D2: Bias due to deviations from intended intervention.

D3: Bias due to missing outcome data.

D4: Bias in measurement of the outcome.

D5: Bias in selection of the reported result.

Judgement:

: Low risk of bias

: some concerns risk of bias

: High risk of bias

**S2 Fig. Network** **plot and league plot of the network estimates for comparing once-weekly insulin with once-weekly Fc for the risk of any adverse event.**


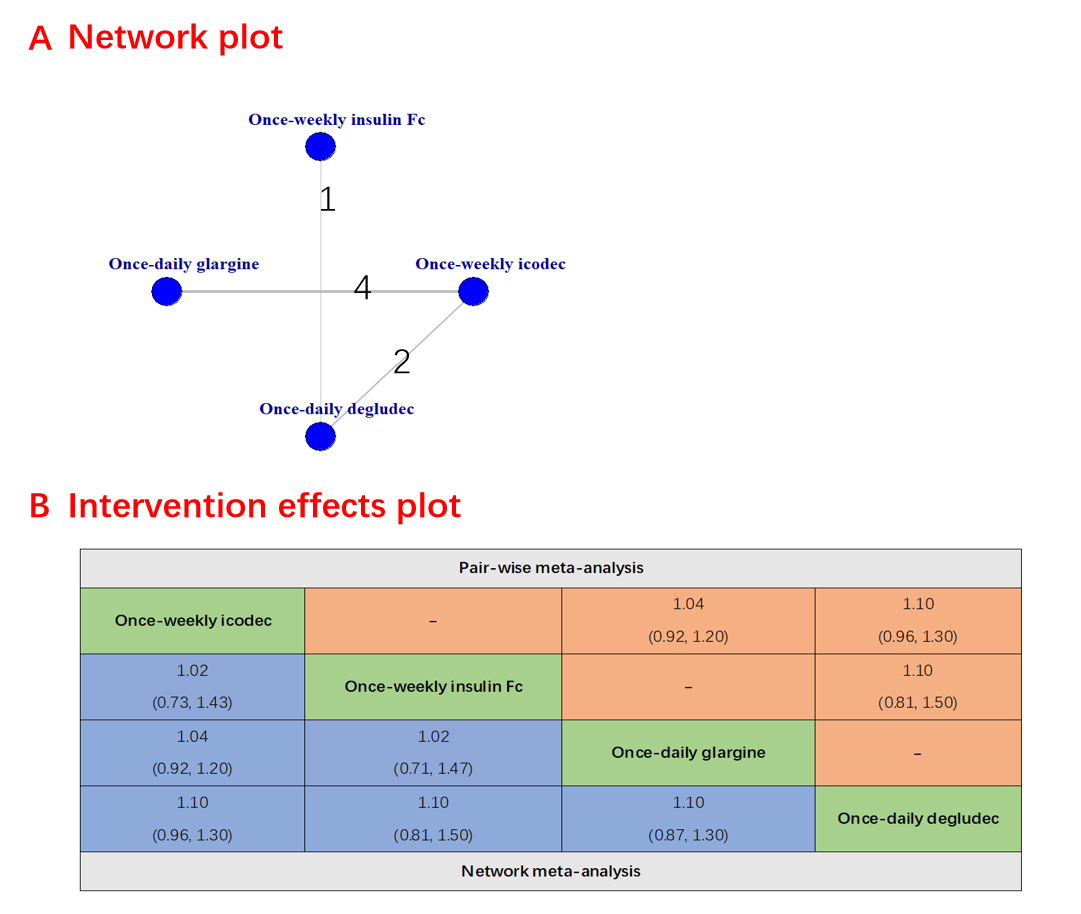


**S3 Fig. Network plot and league plot of the network estimates for comparing once-weekly insulin with once-weekly Fc for the risk of serious adverse event.**

**
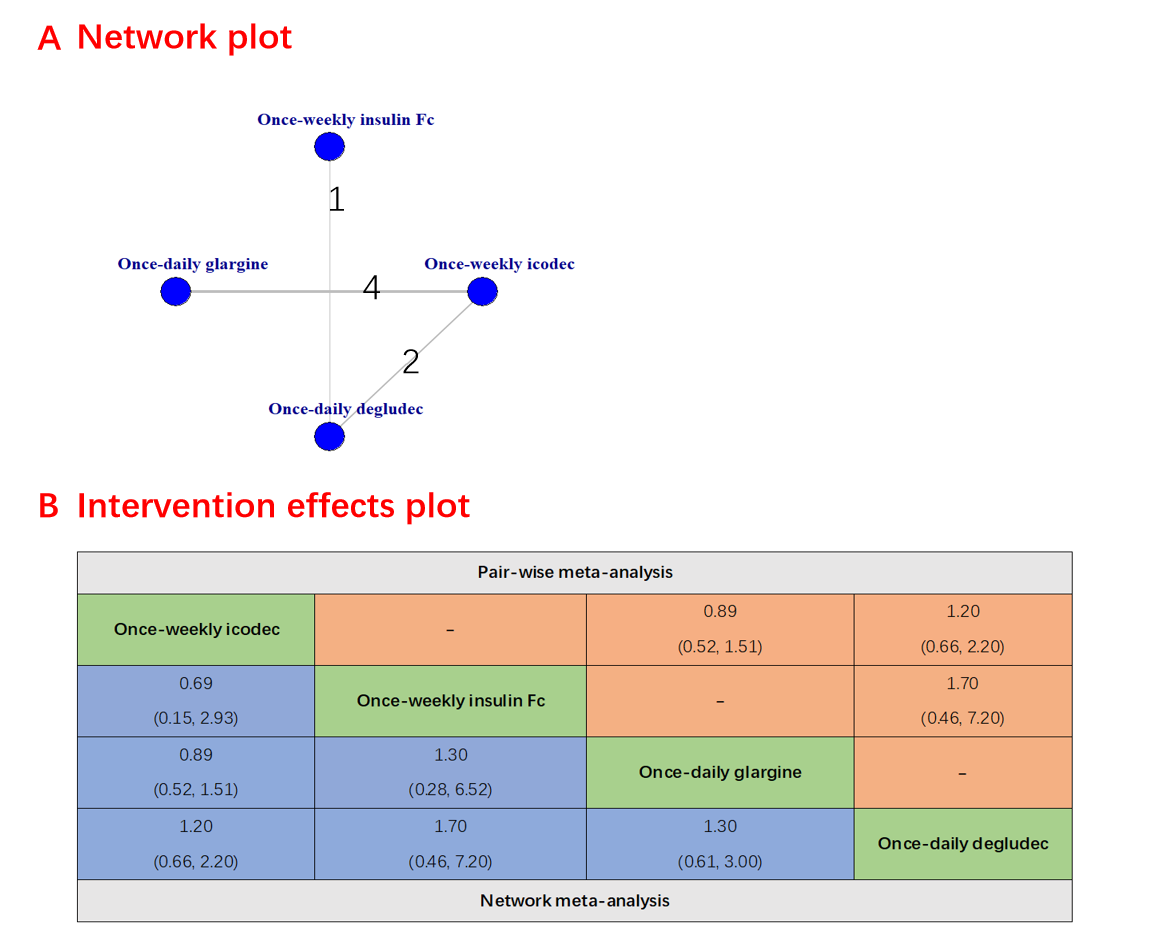
**

**S4 Fig. Network plot and league plot of the network estimates for comparing once-weekly insulin with once-weekly Fc for the risk of any injection-site reaction.**

**
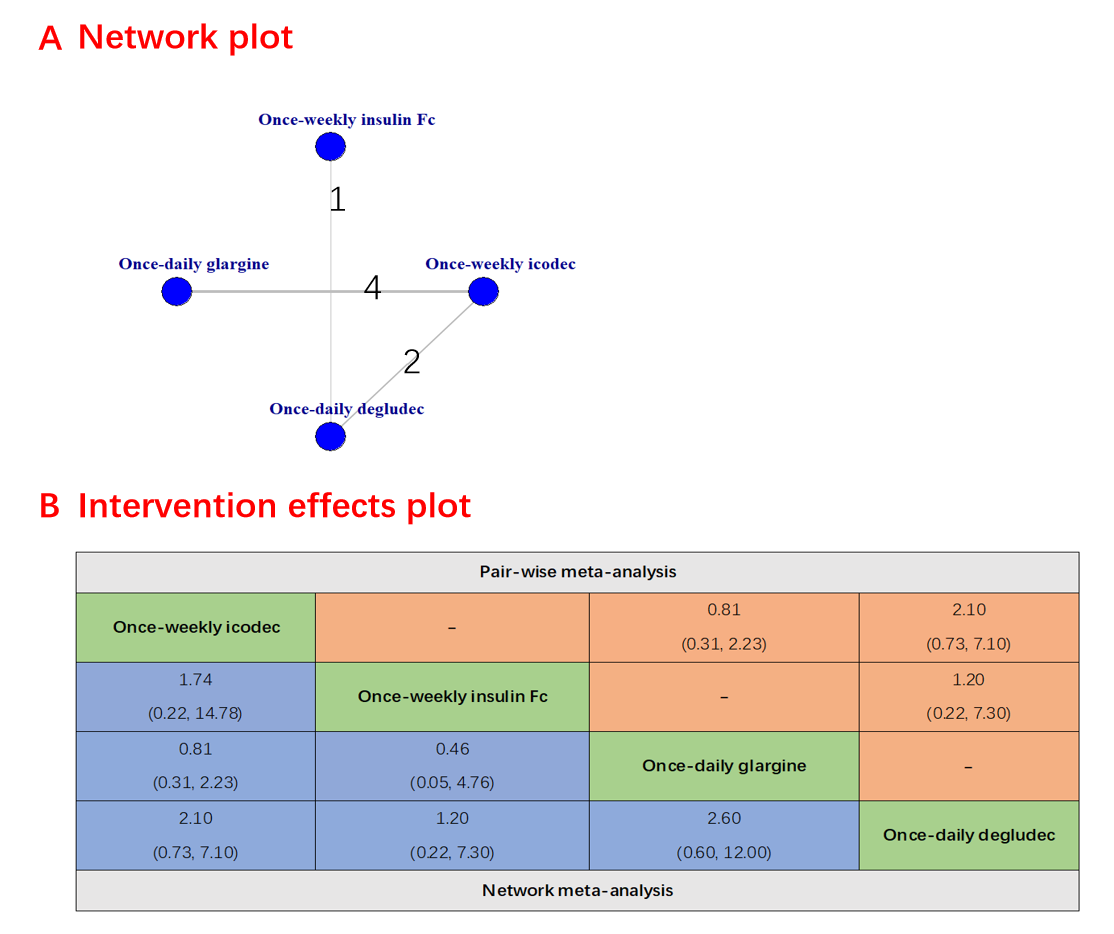
**
