# Supplementary material for: Efficacy and Safety of Once-Weekly Insulin Regimes on Glycemic Control for Type 2 Diabetes: A Systematic Review and Network Meta-analysis
Source: Diabetol Metab Syndr. 2024 Jan 3;16:3. doi: 10.1186/s13098-023-01240-5 (PMC10763463; doi:10.1186/s13098-023-01240-5)
Supplement: Supplementary file 3 — Supplementary Material 3 [file 13098_2023_1240_MOESM3_ESM.docx]

Dear editor-in-chief,

We confirm that none of the material in this manuscript has been published or is under consideration for publication elsewhere. We confirm that any actual, potential, financial, or ethical problems or conflicts of interest are unrelated to the submitted manuscript. All authors read and abided by the statement of ethical standards for manuscripts submitted to the *Diabetology & Metabolic Syndrome.*

Yours sincerely

Fang Fang Professor

West China Hospital, Sichuan University, No. 37, Guo Xue Xiang, Chengdu, Sichuan, 610041, China. **Email: fangfang01@scu.edu.cn**
